# Racial bias in implicit danger associations generalizes to older male targets

**DOI:** 10.1371/journal.pone.0197398

**Published:** 2018-06-06

**Authors:** Gustav J. W. Lundberg, Rebecca Neel, Bethany Lassetter, Andrew R. Todd

**Affiliations:** 1 Department of Psychology, New York University, New York, NY, United States of America; 2 Department of Psychological and Brain Sciences, University of Iowa, Iowa City, IA, United States of America; 3 Department of Psychology, University of California, Davis, Davis, CA, United States of America; Boston Children's Hospital / Harvard Medical School, UNITED STATES

## Abstract

Across two experiments, we examined whether implicit stereotypes linking younger (~28-year-old) Black versus White men with violence and criminality extend to older (~68-year-old) Black versus White men. In Experiment 1, participants completed a sequential priming task wherein they categorized objects as guns or tools after seeing briefly-presented facial images of men who varied in age (younger versus older) and race (Black versus White). In Experiment 2, we used different face primes of younger and older Black and White men, and participants categorized words as ‘threatening’ or ‘safe.’ Results consistently revealed robust racial biases in object and word identification: Dangerous objects and words were identified more easily (faster response times, lower error rates), and non-dangerous objects and words were identified less easily, after seeing Black face primes than after seeing White face primes. Process dissociation procedure analyses, which aim to isolate the unique contributions of automatic and controlled processes to task performance, further indicated that these effects were driven entirely by racial biases in automatic processing. In neither experiment did prime age moderate racial bias, suggesting that the implicit danger associations commonly evoked by younger Black versus White men appear to generalize to older Black versus White men.

## Introduction

On July 16, 2009, Harvard University professor Henry Louis Gates, Jr., returned home to find the lock on his front door jammed. By the time Gates had entered his house through a back door, a neighbor had already called the Cambridge, Massachusetts, police to report a potential burglary in progress. The subsequent chain of events ultimately led to Gates’ being arrested and charged with disorderly conduct. Gates was 59 years old at the time, walked with the help of a cane, and looked like the prototypical image of a tenured academic in his manner of dress [1–3]. As a physically unimposing older man, it stands to reason that Gates would be unlikely to be mistaken for a criminal. Gates, however, is also African American, and deep-seated racial stereotypes associating Black men with violence and criminality persist in the United States. Gates’ arrest thus raises a broader question: Do pervasive associations more strongly linking young Black men than young White men with danger extend to older Black versus White men, or do these racially-biased stereotypes simply not apply to men of advanced age? This question was the focus of the current research.

Young Black men are commonly viewed as hostile, aggressive, and posing an imminent threat to the physical safety of those they encounter [4, 5]. These stereotypes can bias social judgment and decision making in numerous ways. For example, relative to young White men, young Black men are more likely to be misidentified and misremembered as angry [6–9]. Innocuous objects (e.g., tools, cellphones) are also more likely to be mistaken as threatening ones (e.g., guns) in the hands of–or even in the mere presence of–young Black men versus young White men [10–14] (but see ref. [15]).

Despite strong links between Black men and danger, stereotypes of older adults may moderate these associations. Men of advanced age are, in fact, less likely to commit acts of violence than are younger men [16]. Furthermore, older adults are typically viewed as not wanting to harm others [17], and normal age-related declines in muscle mass and strength imply a reduced capacity to do so [18]. Thus, even in cases in which they may explicitly intend to inflict harm, older adults may be seen as not competent or limber enough to pose a credible threat [17]. This line of reasoning suggests that danger-based racial biases may be weaker in response to older adults, with such stereotypes applying more strongly to young Black men versus non-Black men [19]. Indeed, some evidence indicates that older Black men are viewed as more communal than older White men [20]; insofar as communal people tend to be viewed as benevolent and likeable [21], it stands to reason that they might also be unlikely to be viewed as dangerous. Another study found that, whereas participants tended to recognize anger more readily on the faces of younger Black versus White men, this pattern of racial bias in emotion recognition reversed when participants viewed older men’s faces [22]. Together, these findings suggest that, insofar as older Black adults are seen as deviating from the prototype of the category “Black” (cf. ref. [23]), danger-based racial biases commonly observed in response to young Black and White men may not emerge as strongly–or at all–in response to older Black and White men.

An alternative possibility–that danger-based racial biases transcend age–is suggested by evidence that many of the same stereotypic associations commonly evoked by ~25-year-old Black versus White men may be evoked just as readily by ~5-year-old Black versus White boys [24, 25] (see also refs. [26–28]). Further supporting this possibility is work that has reported racial bias in explicit ratings of agentic qualities for older Black and White adults [29]. More germane to the current investigation is research directly linking older Black versus White men with dangerous objects. In one study, for example, undergraduates completed a sequential priming task–the weapon identification task [14]–in which they categorized objects as guns or tools after brief presentations of facial images of Black and White men who varied in age (younger versus older) [30]. While completing this task, half the participants kept a mental tally of how many Black men and how many White men they saw, thereby increasing the salience of the race of the face primes. Remaining participants kept track of how many younger men and how many older men they saw, thereby increasing the salience of the age of the face primes. As is commonly observed with the weapon identification task, gun categorizations were easier and tool categorizations were more difficult after seeing faces of Black versus White men. This pattern of racial bias linking Black men with guns, which only emerged when participants kept track of the race of the face primes but not when they kept track of the age of the face primes (see also ref. [31]), was not moderated by prime age. That is, when prime race was made salient during the task, participants displayed a robust pattern of racial bias in weapon identification, regardless of prime age. These results suggest that implicit associations linking young Black versus White men with dangerous objects may extend to older Black and White men. Because this prior work did not include a condition in which neither prime race nor prime age had been made salient during the task, however, it is unclear whether the observed pattern of generalization of racial bias across prime age would emerge in the absence of an experimental manipulation that was specifically designed to draw attention to the race of the face primes. Examining this possibility was the focus of the experiments reported here.

## Overview of experiments

We conducted two experiments testing whether implicit racial stereotypes of young Black versus White men as dangerous generalize to older Black versus White men. In Experiment 1, we used a weapon identification task [14] that was similar to the one used by Jones and Fazio [30]. Participants classified objects as guns or tools after briefly-presented male faces that varied in race (Black versus White) and age (younger [perceived age: ~28 years] versus older [perceived age: ~68 years]). Experiment 2 was a conceptual replication, with two primary changes. First, instead of classifying objects as guns or tools, participants classified words as ‘threatening’ or ‘safe’ [25, 32]. Second, to protect against the possibility that effects observed in Experiment 1 could be attributed to idiosyncrasies of the specific faces of Black and White men that served as primes, and to ensure adequate stimulus sampling [33], we included a new set of face primes.

In both experiments, our primary metrics of interest were response times (RTs) and error rates in object and word categorization, as is common in research using sequential priming tasks [34]. For both metrics, we were interested in (i) identifying potential differences in object and word identification following Black face primes versus White face primes (i.e., racial bias) and (ii) determining whether the magnitude of this racial bias differed as a function of prime age. One problem with this approach (sometimes called the “task-dissociation” approach), however, is that it equates a behavioral effect on an implicit/indirect measure (e.g., misidentifying tools as guns more often after seeing faces of Black men versus White men) with the core construct of interest: “implicit racial bias.” Rather than assuming that implicit/indirect measures (e.g., the weapon identification task) capture only automatic processes and that explicit/direct measures (e.g., self-reported survey items) capture only controlled processes (sometimes called the “process-purity” assumption), we instead assume that both automatic *and* controlled processes contribute to behavioral effects on both types of tasks [14, 35]. Consequently, claims of automaticity in sequential priming measures of racial bias like those used here require isolating the unique contributions of automatic and controlled processes to task performance.

A well-established technique for decomposing automatic and controlled processes in a single behavioral task is the process dissociation procedure (PDP). Originally developed to estimate latent processes that interact to shape behavioral performance on memory tasks [35], variants of the PDP have been used to disentangle component processes underlying performance on implicit/indirect measures in a wide range of other domains, including empathy for pain [36], visual perspective taking [37], moral judgment [38], and, of most relevance for the current work, racial stereotyping [10, 14, 24, 25]. We used the PDP here to quantify the independent contributions of automatic and controlled processes to sequential priming task performance. For both experiments, we report our a priori sample size rationale, as well as all data exclusions, manipulations, and measures.

## Experiment 1

### Method

#### Ethics statement

The Institutional Review Board at the University of Iowa approved this research and the informed consent process (IRB ID#: 201507808). This research was determined to involve “no more than minimal risk.” All participants read a document describing the experimental procedures and provided oral consent prior to participating, and they read a debriefing document at the end of the experimental session.

#### Power and participants

In Experiment 1, we aimed to collect enough data to ensure 90% a priori power to detect a medium-sized effect (η_p_^2^ = .06), settling on a target sample of at least 168 participants [39]. Data were collected until this target number was surpassed. University of Iowa undergraduates (*N* = 193) participated for course credit. A computer malfunction resulted in data loss for 1 participant. Because performance at or below chance can indicate confusion regarding task instructions (e.g., which key mapped to which response), data from 10 additional participants with below-chance accuracy (errors on >50% of trials) on the weapon identification task were excluded from analyses. The final sample comprised 182 participants (115 women, 63 men, and 4 unreported; 141 White, 3 Black, 17 Latinx, 13 Asian, 4 who reported more than one race/ethnicity, and 4 unreported).

#### Procedure and materials

As part of a study purportedly investigating “rapid categorization judgments under distracting conditions,” participants completed a sequential priming task–the weapon identification task [14]–during which they saw two images flash in quick succession. They were instructed to ignore the first distracter image (i.e., the prime), which always displayed a face, and to quickly and accurately categorize the second image (i.e., the target object) as either a gun or a tool by pressing one of two response keys (counter-balanced across participants).

The target objects were 6 images of guns and 6 images of tools (e.g., a wrench) used by Payne [14] and obtained from Keith Payne’s website (http://bkpayne.web.unc.edu/research-materials/). The primes were the same 40 head-and-shoulder photos of younger and older (see below for ratings of perceived age for facial stimuli in both experiments) White and Black men (10 of each category combination) used by Gawronski et al. [31]. These facial images were originally obtained from the Florida Department of Corrections website (http://www.dc.state.fl.us/), which maintains an easily accessible database with information, including photos, for all incarcerated inmates. This database has been used extensively in prior research [40–43], in large part because of its public availability.

To ensure the suitability of these facial images, we conducted a pilot study in which University of Iowa undergraduates (*N* = 266) rated the face primes from Experiments 1 and 2 on perceived age, perceived race, attractiveness, babyfacedness, masculinity, Afrocentricity, and emotional expressiveness. Across experiments, analyses of these data revealed significant group differences tracking the intended prime age and prime race categories. The younger face primes were rated as being younger than the older face primes (*M*s = 27.95 vs. 67.65 years, respectively; *p* < .001). More than 90% of participants rated the White primes as White and the Black primes as Black, whereas close to 0% of participants rated the White primes as Black and the Black primes as White. Other group differences emerged that are largely consistent with the existing literature on ratings of Black and White faces. For example, in line with prior work, faces of Black men were rated as more masculine than were faces of White men (see, e.g., refs. [44–46]), and this racial difference was comparable across age groups. For additional details about the method and results of the pilot study, see S1 Supporting Information.

The sequence of each trial in the weapon identification task was as follows: fixation cross (500 ms), face prime (200 ms), target object (200 ms), and pattern mask (on screen until participants responded, after which the next trial began). A message (*Please respond faster*!) appeared for 1 s if participants did not respond within a 500 ms response deadline, after which the next trial began. Participants completed a total of 288 randomly-ordered experimental trials. Sixteen practice trials preceded the experimental trials.

### Results

For all mixed-effects analyses in both experiments, we tested a model with fixed effects of Prime Age (young = -.5, old = .5), Prime Race (White = -.5, Black = .5), and Target Object/Word (tool/safety = -.5, gun/threatening = .5). In all models, we included random intercepts for prime, target, and participant. Our initial models included random participant slopes of the primary effect of interest, the Prime Race × Target Object interaction, as well as main effect slopes of Prime Race and Target Object; however, the RT model did not converge until we removed the interaction as a participant slope, and the error rate model did not converge until we removed both the interaction and the Prime Race slopes. Thus, the final RT model included random slopes of Target Object/Word and Prime Race for participant, and the final error rate model included a random slope of Target Object/Word for participant.

Preliminary analyses revealed that participant gender did not moderate any of the racial bias effects in either experiment; thus, we collapsed across this variable in our analyses. Additionally, excluding Black participants’ data (Experiment 1: *N* = 3; Experiment 2: *N* = 5) left the results in both experiments unchanged.

#### RTs

Prior to analysis, we excluded trials with errors (incorrect object identifications) and RTs <100 ms. RTs >500 ms were coded as errors, and 500 ms was used as the upper bound for RT inclusion [26]. RTs were log-transformed to reduce positive skew [14], but we report raw RTs to improve the ease of interpretation. Table 1 presents all inferential statistics from the omnibus analyses of RTs for Experiments 1 and 2.

**Table 1:** Summary of fixed effects in mixed-effects models for Prime Race, Prime Age, and target object/word predicting log-transformed response times (Experiments 1 and 2).

	Experiment 1				Experiment 2			
	*B*	*SE*	*t*	*p*	*B*	*SE*	*t*	*p*
Intercept	5.605	.016	361.95	< .001	6.215	.013	482.62	< .001
Prime Race	.000	.003	0.06	.952	-.002	.003	-0.81	.421
Prime Age	.012	.003	4.20	< .001	.008	.002	3.16	.002
Target Object/Word	-.093	.025	-3.69	.003	-.009	.019	-0.48	.644
Prime Race × Prime Age	.007	.006	1.17	.250	.009	.005	1.75	.080
Prime Race × Target Object/Word	-.073	.006	-13.06	< .001	-.024	.005	-4.99	< .001
Prime Age × Target Object/Word	-.013	.006	-2.32	.020	-.002	.005	-0.43	.665
Prime Race × Prime Age × Target Object/Word	.009	.011	0.78	.433	.010	.010	0.98	.330

A mixed-effects model on the RTs yielded a significant Prime Race × Target Object interaction indicative of racial bias, *b* = -.073, *se* = .006, CI_95%_ [-.084, -.062], *t* = -13.06, *p <* .001. Decomposing this interaction revealed that participants identified guns more quickly after Black primes versus White primes, *b* = -.037, *se* = .005, CI_95%_ [-.047, -.027], *t* = -7.08, *p <* .001, whereas they identified tools more quickly after White primes versus Black primes, *b* = .038, *se* = .005, CI_95%_ [.028, .048], *t* = 7.39, *p <* .001. The Prime Age × Target Object interaction was also significant, *b* = -.013, *se* = .006, CI_95%_ [-.024, -.002], *t* = -2.32, *p =* .020. Decomposing this interaction revealed that, whereas participants identified guns no more quickly after younger primes versus older primes, *b* = .006, *se* = .005, CI_95%_ [-.003, .015], *t* = 1.25, *p* = .22, they identified tools more quickly after younger primes versus older primes, *b* = .019, *se* = .004, CI_95%_ [.011, .027], *t* = 4.47, *p <* .001. The Prime Age × Prime Race × Target Object interaction was not significant, *b* = .009, *se* = .011, CI_95%_ [-.013, .031], *t* = 0.78, *p =* .433, suggesting a comparable pattern of racial bias in object identification across prime age.

**Fig 1:**
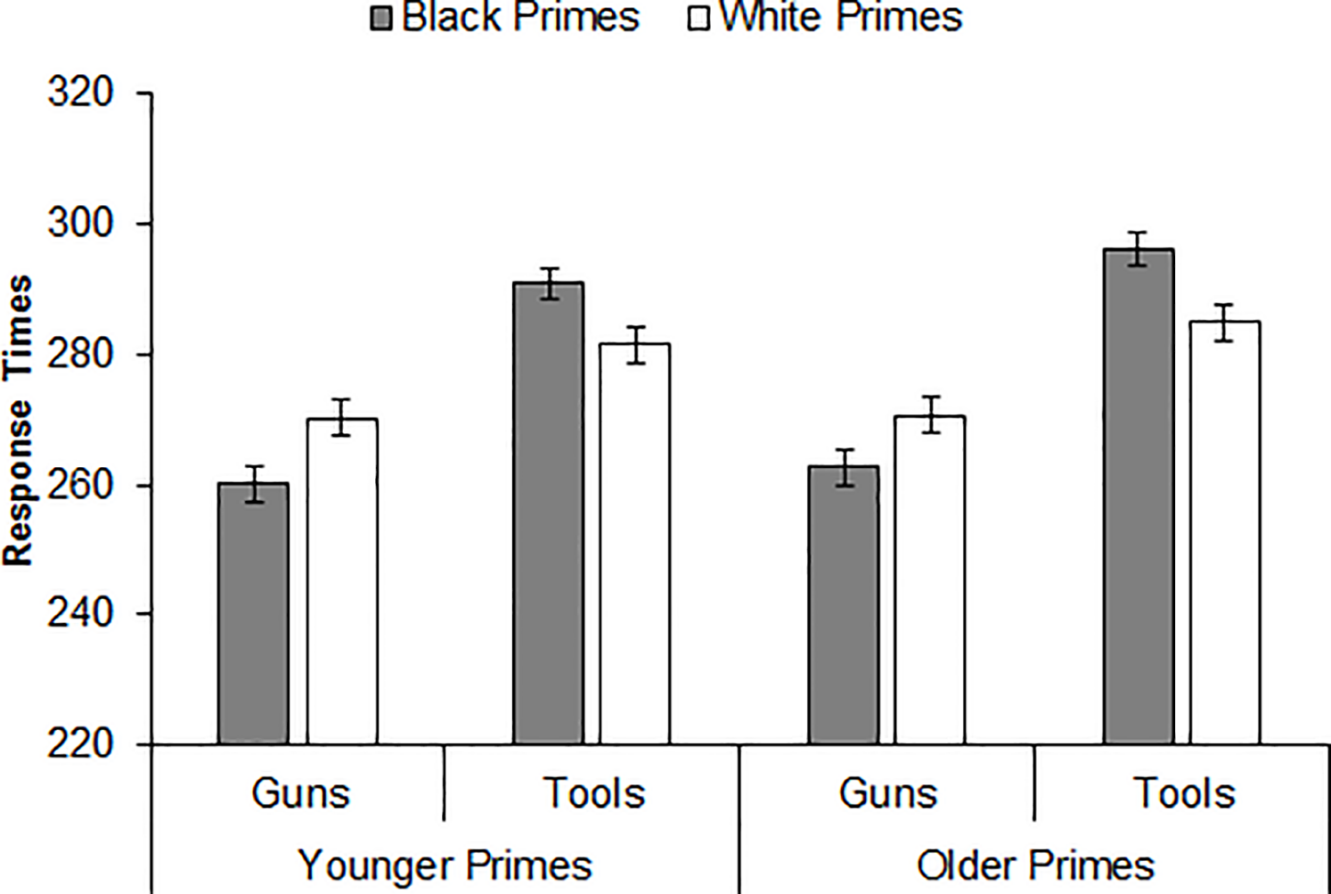
Mean response times (in milliseconds) for gun and tool identifications by prime race and prime age (Experiment 1).

To test the strength of the evidence that younger and older primes elicited comparable racial bias against an alternative hypothesis that racial bias was weaker after older primes versus younger primes, we created indices of racial bias separately for younger primes and older primes as follows [24, 25, 47]: (White face–gun trials minus Black face–gun trials) + (Black face–tool trials minus White face–tool trials). We then used Rouder’s Bayes-factor calculator [48] for paired samples (http://pcl.missouri.edu/bayesfactor) to estimate the Jeffreys-Zellner-Siow (JZS) Bayes factor for the comparison of racial bias after younger primes versus older primes; the scale *r* was set to the default of .707. The JZS Bayes factor indexes the strength of evidence in favor of a null hypothesis over an alternative hypothesis. This analysis favored no difference in racial bias across prime age: JZS Bayes factor = 11.06 (i.e., no difference between younger primes and older primes was about eleven times more likely than a difference). Indeed, as depicted in Fig 1, the Prime Race × Target Object interaction indicative of racial bias was significant after both younger primes, *b* = -.077, *se* = .008, CI_95%_ [-.093, -.061], *t* = -9.69, *p <* .001, and older primes (note that convergence of the model with only older primes required dropping all slopes for participant), *b* = -.068, *se* = .008, CI_95%_ [-.083, -.052], *t* = -8.59, *p <* .001.

### Error rates

Table 2 presents all inferential statistics from the omnibus analyses of error rates for Experiments 1 and 2.

**Table 2:** Summary of fixed effects in binomial mixed-effects models for Prime Race, Prime Age, and target object/word predicting error rates (Experiments 1 and 2).

	Experiment 1				Experiment 2			
	*B*	*SE*	*Z*	*p*	*B*	*SE*	*Z*	*p*
Intercept	-2.050	.103	-19.86	< .001	-1.745	.139	-12.57	< .001
Prime Race	-.025	.027	-0.92	.356	-.015	.026	-0.56	.575
Prime Age	.001	.027	0.02	.983	-.010	.026	-0.38	.701
Target Object/Word	-.007	.175	-0.04	.969	-.347	.262	-1.33	.185
Prime Race × Prime Age	-.092	.054	-1.71	.087	-.014	.053	-0.26	.798
Prime Race × Target Object/Word	-.517	.052	-9.88	< .001	-.290	.051	-5.65	< .001
Prime Age × Target Object/Word	-.057	.052	-1.09	.276	.052	.051	1.01	.313
Prime Race × Prime Age × Target Object/Word	-.060	.105	-0.57	.568	-.031	.102	-0.31	.759

**Fig 2:**
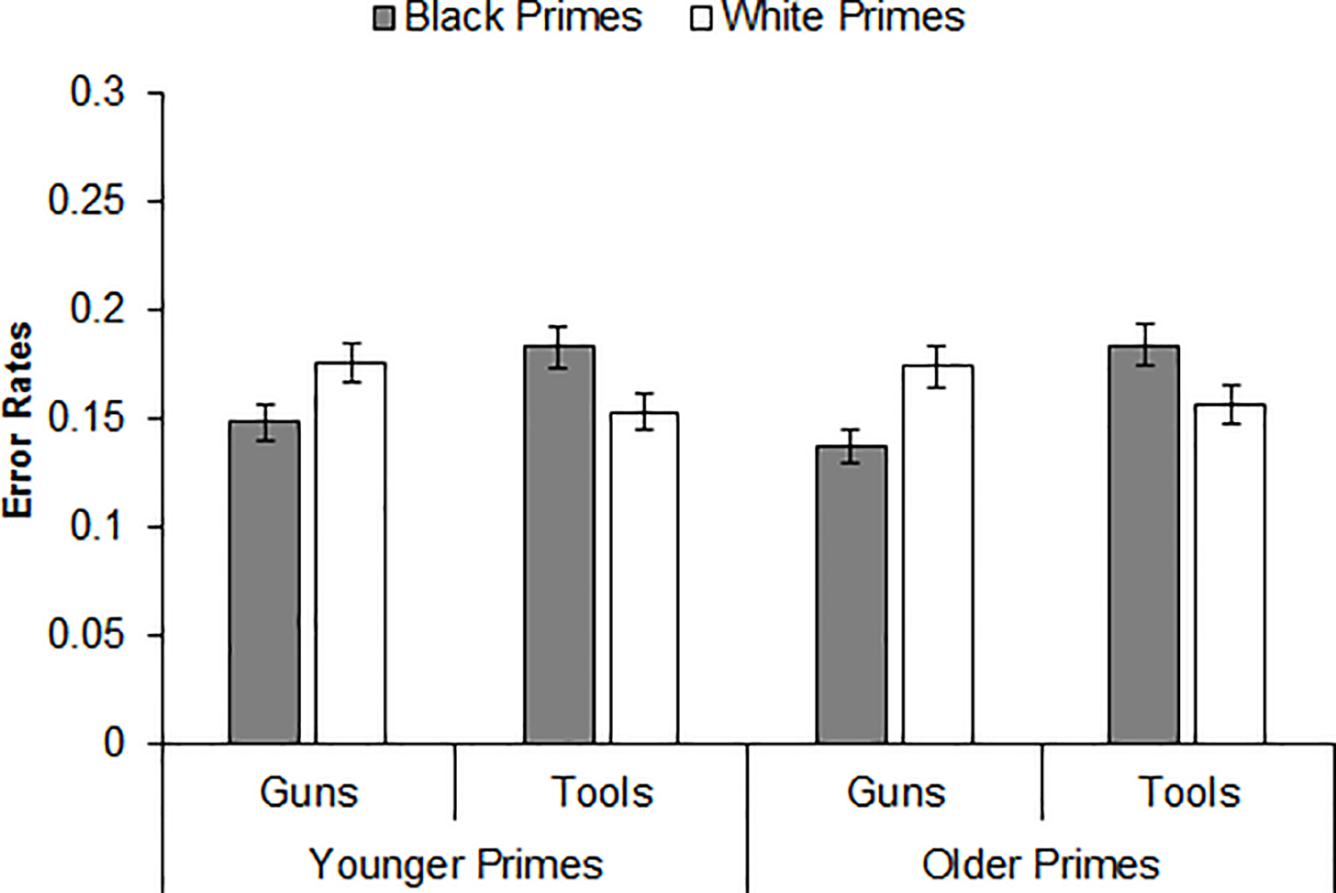
Mean error rates for gun and tool identifications by prime race and prime age (Experiment 1).

A binomial mixed-effects model on the error rates also produced a Prime Race × Target Object interaction, *b* = -.517, *se* = .052, CI_95%_ [-.620, -.415], *Z* = -9.88, *p <* .001. Decomposing this interaction revealed that participants misidentified tools as guns more often after Black primes versus White primes, *b* = .234, *se* = .037, CI_95%_ [.162, .306], *Z* = 6.35, *p <* .001, whereas they misidentified guns as tools more often after White primes versus Black primes, *b* = -.28, *se* = .041, CI_95%_ [-.364, -.203], *Z* = -6.93, *p <* .001. Neither the Prime Age × Target Object interaction, *b* = -.057, *se* = .052, CI_95%_ [-.160, .046], *Z* = -1.09, *p =* .276, nor the Prime Age × Prime Race × Target Object interaction was significant, *b* = -.060, *se* = .105, CI_95%_ [-.265, .145], *Z* = -0.57, *p =* .568, indicating a comparable pattern of object identification across prime age and a comparable pattern of racial bias in object identification across prime age. We computed racial bias for error rates using the same formula as for RTs. The JZS Bayes factors for the difference in racial bias after younger primes versus older primes was 10.51 in favor of the null hypothesis. As depicted in Fig 2, the Prime Race × Target Object interaction indicative of racial bias was again significant after both younger primes, *b* = -.487, *se* = .074, CI_95%_ [-.631, -.342], *Z* = -6.60, *p <* .001, and older primes, *b* = -.548, *se* = .074, CI_95%_ [-.692, -.403], *Z* = -7.42, *p <* .001.

#### PDP estimates

We next conducted PDP analyses to estimate the unique contributions of automatic and controlled processes to task performance. The PDP approach assumes that these processes can be dissociated by creating conditions that place these processes both in concert and in opposition [35]. For example, when a gun appears after a Black face prime, both automatically activated racial bias and accurately identifying the object lead to the same “gun” response (i.e., congruent trials). In contrast, when a tool appears after a Black face prime, automatically activated racial bias favors a “gun” response, but accurately identifying the object favors a “tool” response (i.e., incongruent trials). The critical equations for calculating estimates of controlled and automatic processing are as follows (for the full set of equations, see ref. [49]):

Controlledprocessing=P(correct|congruenttrials)–P(incorrect|incongruenttrials)

Automaticprocessing=P(incorrect|incongruenttrials)/(1−controlledprocessing)

Thus, in the weapon identification task, controlled processing reflects the ability to distinguish guns from tools, independent of response biases. Automatic processing on this task, in contrast, reflects the unintentional biasing influence of the face primes when controlled processing fails (for empirical evidence validating the meaning of these process parameters, see refs. [14, 49]).

Using these equations, we computed estimates of automatic and controlled processing separately for primes of each age–race combination. In cases of perfect performance (i.e., controlled processing = 1), automatic processing is undefined; thus, we applied an adjustment commonly used in signal-detection analyses (see ref. [50] for details). Because negative estimates of controlled processing violate assumptions of PDP that parameter estimates range from 0 to 1 [35], we replaced such instances with a value of 0 [24, 51]; however, retaining the original (negative) estimates produced nearly identical results. PDP estimates are calculated across stimuli for each participant; thus, ANOVAs are appropriate. Because our effect size estimates, η_p_^2^, were computed from ANOVA *F*-values, we report CI_90%_ for all significant PDP analysis effects [52].

**Fig 3:**
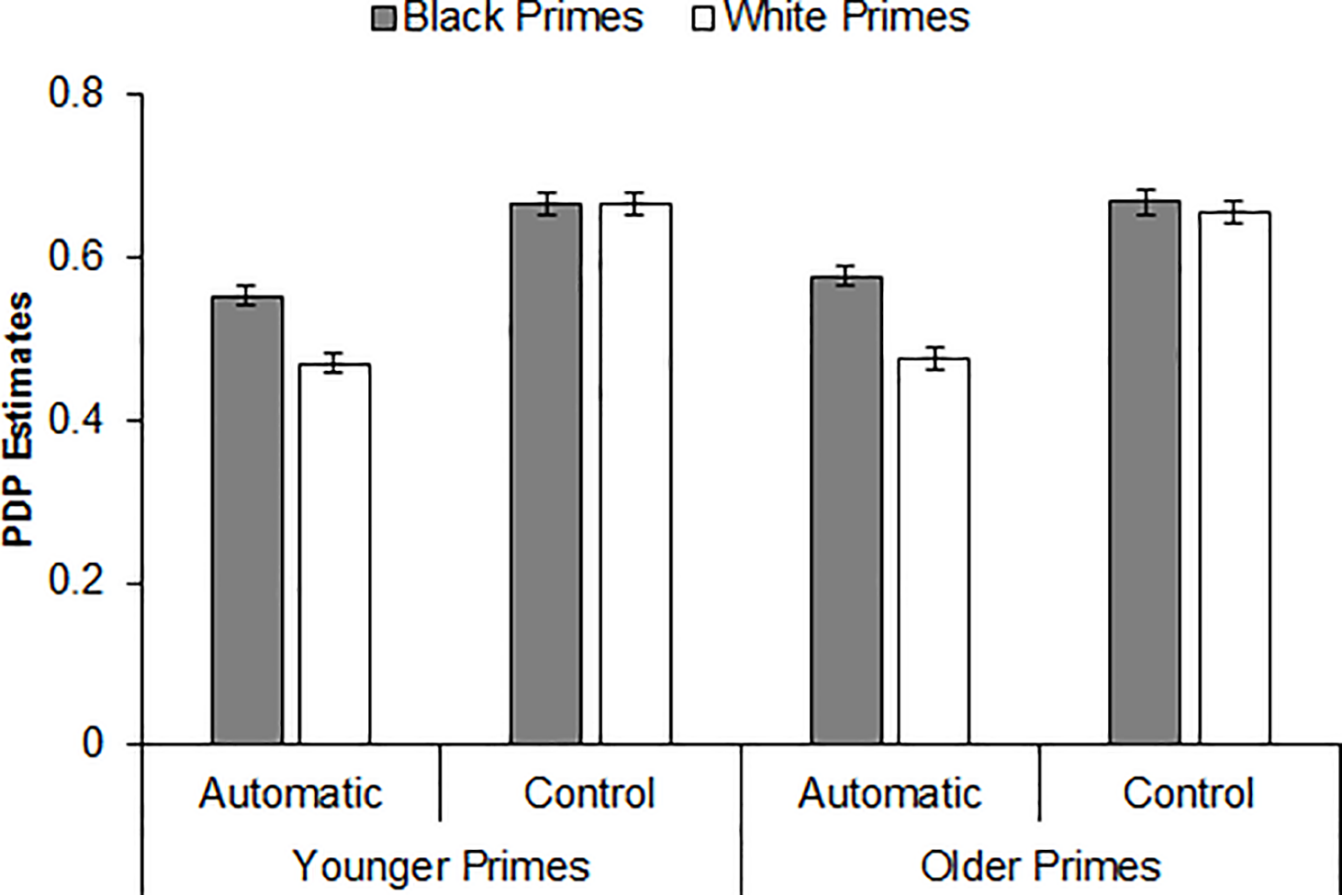
Process dissociation procedure estimates of automatic and controlled processing by prime race and prime age (Experiment 1).

A 2 (Prime Age) × 2 (Prime Race) repeated-measures ANOVA revealed that estimates of automatic processing were greater for Black primes than for White primes, *F*(1, 181) = 61.82, *p* < .001, η_p_^2^ = .26, CI_90%_ [.16, .33]. Neither the Prime Age main effect, *F*(1, 181) = 1.56, *p* = .213, η_p_^2^ < .01, nor the Prime Age × Prime Race interaction was significant (*F* < 1, *p* = .443, η_p_^2^ < .01), indicating a comparable pattern of automatic processing across prime age and a comparable pattern of racial bias in automatic processing across prime age. Comparing racial bias in estimates of automatic processing for younger primes versus older primes produced a JZS Bayes factor of 9.04 in favor of the null hypothesis. Indeed, as depicted in Fig 3, the Prime Race main effect emerged for both younger primes, *F*(1, 181) = 22.20, *p* < .001, η_p_^2^ = .11, CI_90%_ [.04, .18], and older primes, *F*(1, 181) = 40.32, *p* < .001, η_p_^2^ = .18, CI_90%_ [.10, .26]. An identical analysis on the estimates of controlled processing yielded no significant effects (*F*s < 1.14, *p*s > .286, η_p_^2^s < .01). These results indicate that the pattern of racial bias described above, as measured indirectly via the weapon identification task, was driven entirely by differences in automatic processing (and not controlled processing) across prime race.

## Experiment 2

In Experiment 1, participants identified guns more easily and tools less easily following Black versus White face primes. This pattern of racial bias, which emerged in analyses of both RTs and error rates, was comparable across younger and older primes. Furthermore, to avoid the “process purity” assumption that implicit/indirect measures like the weapon identification task capture only a single process (i.e., automatic racial bias), we ran PDP analyses to isolate the unique contributions of automatic and controlled processes to task performance. These analyses further revealed that the behavioral effects described above reflect racial biases in automatic processing. Although these findings support the hypothesis that danger-based racial biases commonly evoked by younger men generalize to older men, it is possible that the racial bias observed here reflects perceived racial differences in access to guns [17] rather than semantic associations linking Black versus White men with danger. Thus, in Experiment 2, we used a sequential priming task that entailed classifying words as ‘threatening’ or ‘safe’ [25, 32] rather than classifying objects as guns or tools. Additionally, to ensure adequate stimulus sampling and thereby protect against the possibility that the results of Experiment 1 can be attributed to idiosyncrasies of the specific face primes [33], we used a new, larger set of facial images of younger and older Black and White men as primes. Based on the results of Experiment 1, we predicted that seeing faces of Black versus White men, regardless of their age, would facilitate the categorization of words connoting threat and would hinder the categorization of words connoting safety.

### Method

#### Power and participants

We determined our target sample size in Experiment 2 using the same criterion as in Experiment 1. Data were collected until this target number was reached. University of Iowa undergraduates (*N* = 168) participated for course credit. We excluded data from 8 participants with below-chance accuracy (errors on >50% of trials) on the weapon identification task, leaving a final sample of 160 participants (103 women, 56 men, and 1 unreported; 127 White, 5 Black, 9 Latinx, 9 Asian, 9 who reported more than one race/ethnicity, and 1 unreported).

#### Procedure and materials

Experiment 2 was identical to Experiment 1, but with three changes. First, instead of completing a weapon identification task, participants completed a sequential priming task [24, 31] in which they classified words as ‘threatening’ (*violent*, *dangerous*, *hostile*, *aggressive*, *criminal*, and *threatening*) or ‘safe’ (*innocent*, *harmless*, *friendly*, *trustworthy*, *peaceful*, and *safe*). Second, we increased the response deadline from 500 ms to 750 ms to account for the increased difficulty of categorizing words versus objects [53]. Third, we selected a new set of 80 facial images of younger and older Black and White men (20 of each category combination) from the same database used in Experiment 1 (for additional details about our image selection procedure, see S2 Supporting Information). As in Experiment 1, participants completed a total of 288 randomly-ordered experimental trials, which were preceded by 16 practice trials.

### Results

#### RTs

RT data were prepared and analyzed as in Experiment 1. Prior to analysis, we excluded trials with errors (incorrect word identifications) and RTs <100 ms. RTs >750 ms were coded as errors, and 750 ms was used as the upper bound for RT inclusion. RTs were log-transformed to reduce positive skew, but we report raw RTs to improve the ease of interpretation.

**Fig 4:**
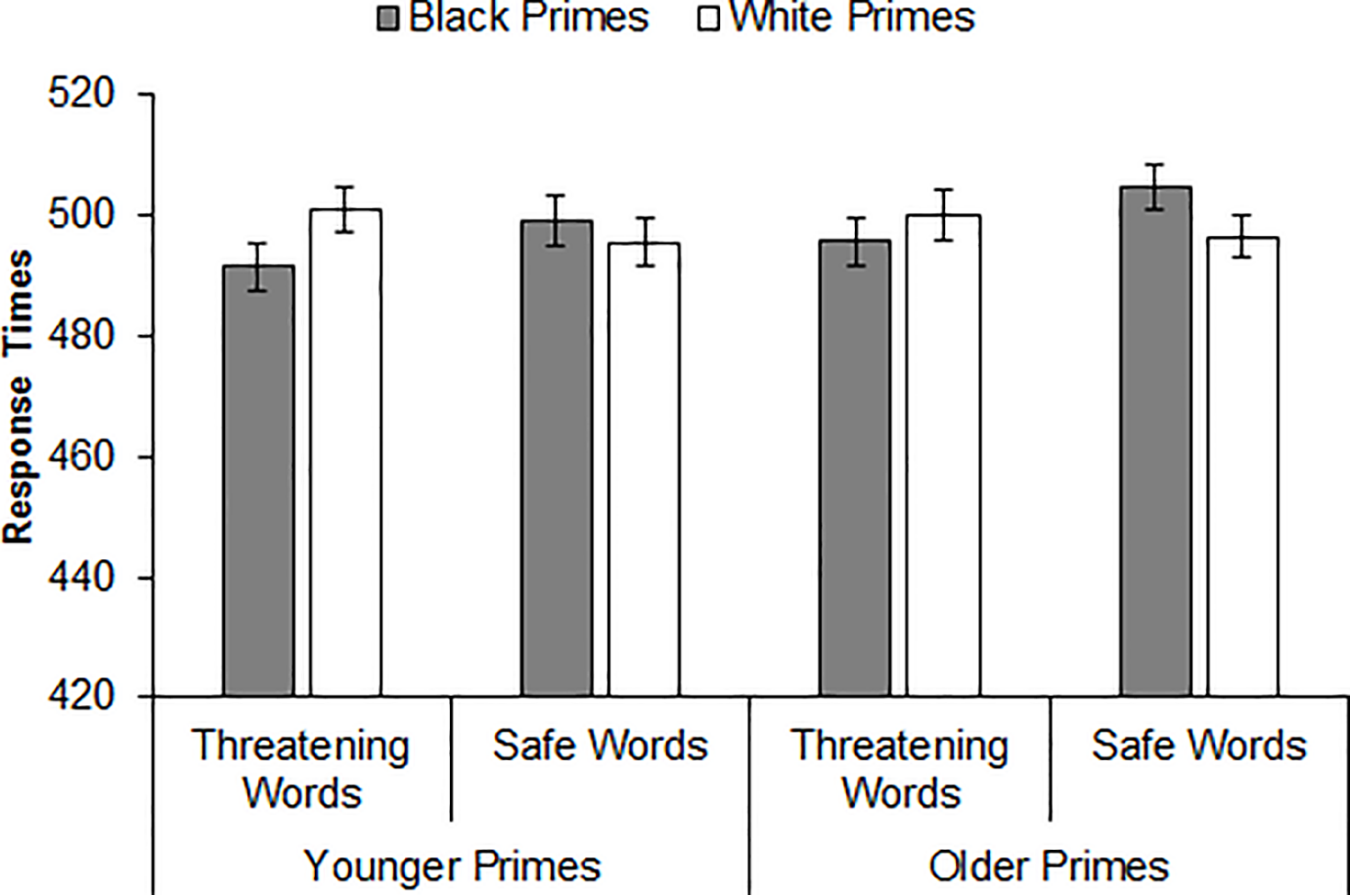
Mean response times (in milliseconds) for threatening and safe word identifications by prime race and prime age (Experiment 2).

This analysis yielded a significant Prime Race × Target Word interaction, *b* = -.024, *se* = .005, CI_95%_ [-.034, -.015], *t* = -4.99, *p <* .001. Decomposing this interaction revealed that participants identified threatening words more quickly after Black primes versus White primes, *b* = -.014, *se* = .004, CI_95%_ [-.023, -.006], *t* = -3.38, *p <* .001, whereas they identified safe words more quickly after White primes versus Black primes, *b* = .010, *se* = .005, CI_95%_ [.001, .019], *t* = 2.16, *p =* .034. Neither the Prime Age × Target Word interaction, *b* = -.002, *se* = .005, CI_95%_ [-.012, .007], *t* = -0.43, *p* = .665, nor the Prime Age × Prime Race × Target Word interaction was significant, *b* = .010, *se* = .010, CI_95%_ [-.010, .029], *t* = 0.98, *p* = .330 (JZS Bayes factor = 10.85 in favor of the null hypothesis), suggesting a comparable pattern of word identification across prime age and a comparable pattern of racial bias in word identification across prime age. As depicted in Fig 4, the Prime Race × Target Word interaction indicative of racial bias was significant after both younger primes, *b* = -.029, *se* = .007, CI_95%_ [-.042, -.015], *t* = 4.10, *p* < .001, and older primes *b* = -.020, *se* = .007, CI_95%_ [-.033, -.006], *t* = 2.91, *p* = .004.

#### Error rates

**Fig 5:**
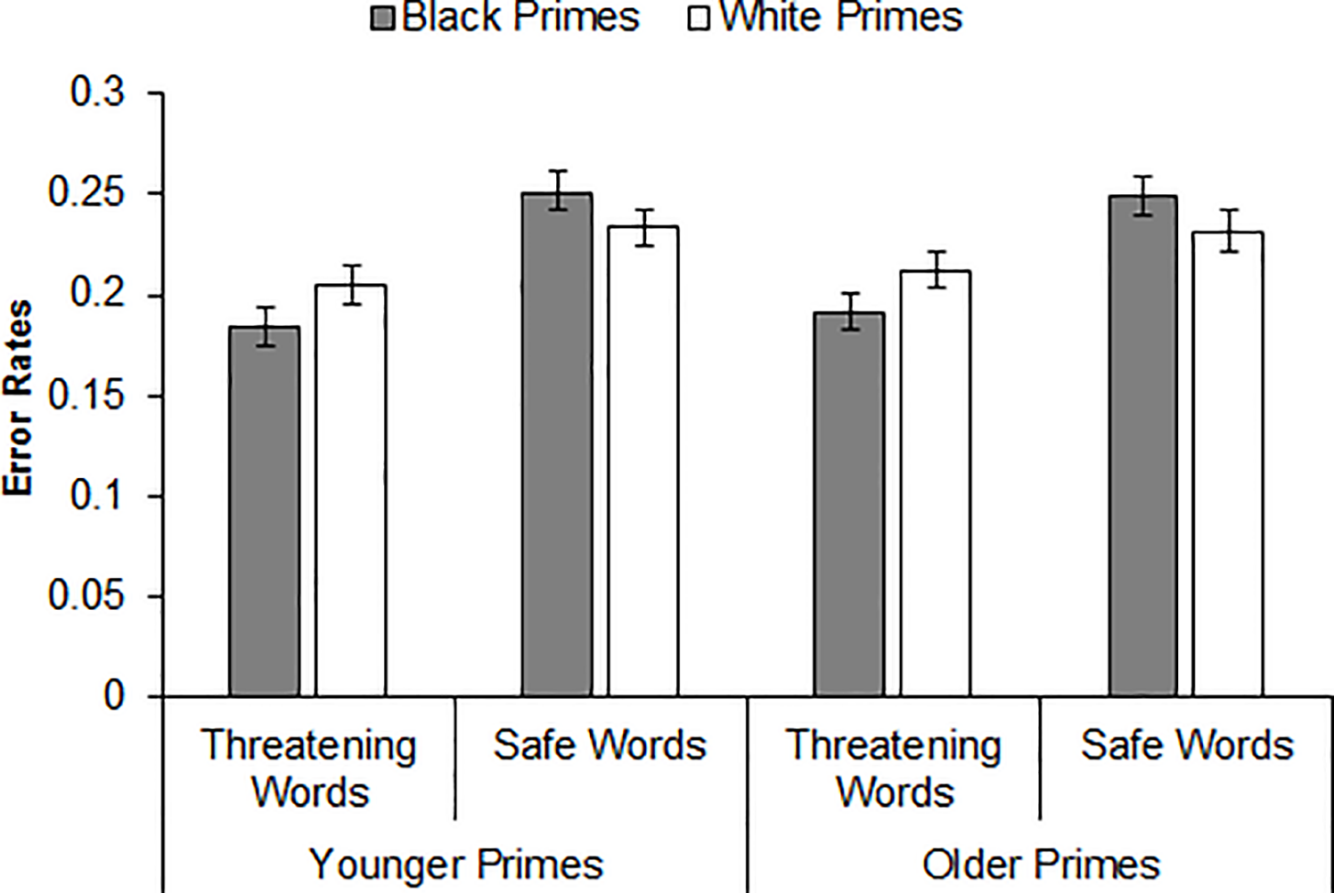
Mean error rates for threatening and safe word identifications by prime race and prime age (Experiment 2).

A Prime Race × Target Word interaction also emerged in an analysis on the error rates, *b* = -.290, *se* = .051, CI_95%_ [-.390, -.189], *Z* = -5.65, *p <* .001. Decomposing this interaction revealed that participants misidentified safe words as threatening more often after Black primes versus White primes, *b* = .130, *se* = .036, CI_95%_ [.060, .201], *Z* = 3.62, *p <* .001, whereas they misidentified threatening words as safe more often after White primes versus Black primes, *b* = -.16, *se* = .042, CI_95%_ [-.243, -.077], *Z* = -3.77, *p <* .001. As was the case with the RT analyses, neither the Prime Age × Target Word interaction, *b* = .052, *se* = .051, CI_95%_ [-.049, .152], *Z* = 1.01, *p =* .313, nor the Prime Age × Prime Race × Target Word interaction was significant, *b* = -.031, *se* = .102, CI_95%_ [-.232, .169], *Z* = -0.31, *p =* .76 (JZS Bayes factor = 11.34 in favor of the null hypothesis), again indicating a comparable pattern of word identification across prime age and a comparable pattern of racial bias in word identification across prime age. As depicted in Fig 5, the Prime Race × Target Word interaction indicative of racial bias was again significant after both younger primes, *b* = -.275, *se* = .072, CI_95%_ [-.417, -.134], *Z* = -3.81, *p <* .001, and older primes, *b* = -.304, *se* = .072, CI_95%_ [-.446, -.163], *Z* = -4.22, *p <* .001.

#### PDP estimates

**Fig 6:**
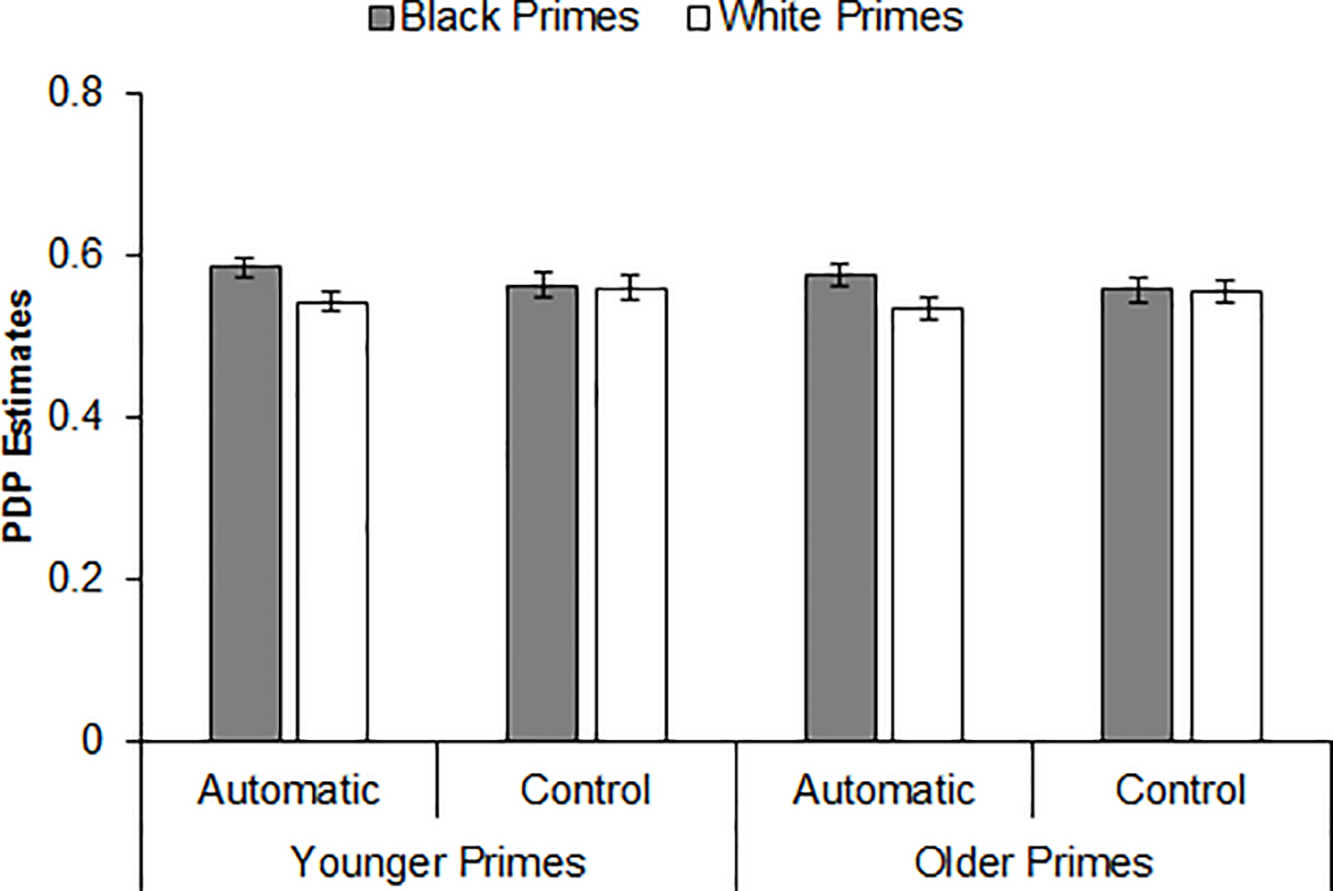
Process dissociation procedure estimates of automatic and controlled processing by prime race and prime age (Experiment 2).

As in Experiment 1, we next conducted PDP analyses to estimate the unique contributions of automatic and controlled processes to task performance. A 2 (Prime Age) × 2 (Prime Race) repeated-measures ANOVA revealed that estimates of automatic processing were greater for Black primes versus White primes, *F*(1, 159) = 12.38, *p* = .001, η_p_^2^ = .07, CI_90%_ [.02, .14]. Neither the Prime Age main effect (*F* < 1, *p* = .350, η_p_^2^ < .01), nor the Prime Age × Prime Race interaction was significant (*F* < 1, *p* = .946, η_p_^2^ < .01; JZS Bayes factor = 11.32 in favor of the null hypothesis), indicating a comparable pattern of automatic processing across prime age and a comparable pattern of racial bias in automatic processing across prime age. As depicted in Fig 6, the Prime Race main effect emerged for both younger primes, *F*(1, 159) = 8.50, *p* = .004, η_p_^2^ = .05, CI_90%_ [.01, .12], and older primes, *F*(1, 159) = 6.22, *p* = .014, η_p_^2^ = .04, CI_90%_ [.004, .10]. An identical analysis on the estimates of controlled processing yielded no significant effects (*F*s < 1, *p*s > .499, η_p_^2^s < .01). These results indicate that, much like in Experiment 1, the racial bias in word identification described above was driven entirely by differences in automatic processing across prime race.

## Discussion

In two experiments, we investigated whether implicit racial stereotypes linking danger more strongly to young Black men than to White men extend to older Black versus White men. Results from both experiments revealed that seeing briefly-presented facial images of Black men, relative to seeing facial images of White men, facilitated the identification of danger-related objects and concepts. This pattern of racial bias was comparable for images of younger and older men and was robust across two different sequential priming paradigms: Both a weapon identification task (Experiment 1) and a word categorization task (Experiment 2) revealed evidence of race-biased danger associations. Process dissociation procedure analyses, which aim to isolate the unique contributions of automatic and controlled processes to sequential priming task performance, further revealed that these effects were driven entirely by racial biases in automatic processing. These results, in conjunction with findings from prior work [24, 25, 28, 31, 32], suggest that implicit biases linking Black men versus White men with danger appear to generalize to male targets ranging in age from youth to older adulthood.

### Limitations and future research directions

We acknowledge several limitations of this work, each of which suggests potential directions for future research. In both experiments, our participant samples comprised mostly White college students, which raises questions about whether these findings will generalize to non-student samples and participants of other races/ethnicities. Additionally, in both experiments, we exclusively used facial images that were taken from the Florida prison database. Our work is not the first to do so. Indeed, because of its public availability and the relative degree of experimental control it affords (e.g., head-and-shoulder photographs taken against a blue background), this database has been used in multiple prior investigations of racial bias [31, 40–43]. Nevertheless, it is important to note that all the men whose faces served as primes in our sequential priming tasks were convicted felons, which necessarily constrains the generalizability of our findings. Although we did not inform participants that the images were of convicted felons, nor were there overt cues within the images indicating that this is the case, it is possible that these facial images differ systematically from the general population of older and younger Black and White men, and that the age-transcendent racial biases reported here are restricted to male targets who have been tried and convicted for engaging in actual criminal activity. Future research will be needed to determine whether the current findings hold with other, non-convict facial images of younger and older Black and White men.

A tacit assumption of the current work is that participants reliably encoded both the race and the age of the men depicted in the prime images. Prior studies using neurophysiological measurement (i.e., EEG) have found that people can extract categorical information about both race [54] and age [55] from facial images within the range of the prime durations used here (i.e., 200 ms) and thus suggest that this assumption is warranted. Nevertheless, insofar as merely thinking about crime can direct attention to faces of Black Americans versus White Americans and activate race-related concepts [56], it is possible that completing tasks that entail classifying objects as guns versus tools or classifying words as ‘threatening’ versus ‘safe’ creates a context in which the race of the primes is more salient than the age of the primes.

A related potential limitation that stems from using facial images as primes is that they do not include all the visual cues that typically would be salient when encountering another person. Future research might benefit from the use of full-body images, which may allow participants to extract additional information about a person’s age, thereby potentially providing more ecologically valid tests of racial bias in danger associations. It seems possible, for example, that, insofar as older adults and children are more likely to have a frail or diminutive stature [17], they may be construed as less dangerous than young adults when participants see accurate representations of physique, potentially modulating racial bias.

It is also worth noting that people rarely, if ever, assess the threat that another person poses without considering available contextual information. For example, people are more likely to appraise outgroup men as dangerous when in the dark than in the light [57]. Future research in which participants receive contextual information via background images [58], information from third parties, or other cues may help paint a more complete picture of how perceivers assess and respond to people of different races in these situations.

Another potential direction for future research could be to use prime images that communicate that a target person comes from a certain type of environment. Prior work has found that racial stereotypes often reflect assumptions about whether a person comes from an environment in which resources are scarce or plentiful [59]. People from resource-scarce environments may be stereotyped as trying to acquire others’ resources via coercion. Thus, if participants receive cues indicating that an individual older Black man has ample resources (e.g., he is wearing a business suit; see ref. [60]), the same danger-based racial stereotypes may no longer be as accessible as they were here (but see ref. [30]).

Finally, in each of our experiments, the focal comparison for gauging racial bias toward younger and older Black men was always White men of the same age. It is possible that implicit associations linking younger and older Black men with danger differ in strength depending on the specific racial group that is used for comparison. Future research could test this possibility by including different racial comparison groups (e.g., Asians). Along these same lines, it is important to acknowledge that our findings speak most directly to contexts in which there are men who vary in both race and age. It will be informative for future studies to examine the operation of implicit danger associations in contexts that solely or primarily comprise Black men. It seems possible that older Black men may evoke weaker danger associations than younger Black men in such contexts.

### Conclusion

In sum, our findings suggest that Henry Louis Gates, Jr.’s experience discussed at the outset may not be unique among older Black men in the United States: Old age, like youth, appears to be insufficient to disarm associations linking Black men with danger. Because these associations can be consequential, even deadly, it will be important for future work to identify whether and how they translate into biased behavior.

## Supporting information

S1 Supporting Information Pilot ratings of facial stimuli in Experiments 1 and 2. (DOCX)

S2 Supporting Information Facial stimuli selection procedure in Experiment 2. (DOCX)
