# Supplementary material for: Racial bias in implicit danger associations generalizes to older male targets
Source: PLoS One. 2018 Jun 6;13(6):e0197398. doi: 10.1371/journal.pone.0197398 (PMC5991338; doi:10.1371/journal.pone.0197398)
Supplement: S1 Supporting Information — (DOCX) [file pone.0197398.s001.docx]

S1 Supporting Information

Pilot Ratings of Facial Stimuli in Experiments 1 and 2

# Interpretation of findings

**Categorization of faces on age and race.** As reported in the main text, across experiments, rating data of the faces’ ages and races revealed significant group differences tracking the intended categories. Younger faces (*M* = 27.95 years) were rated as being significantly younger than older faces (*M* = 67.65 years; *p* < .001). More than 90% of participants rated the White faces as White and the Black faces as Black, whereas close to 0% of participants rated the White faces as Black and the Black faces as White.

**Expected group differences.** Rating data revealed a number of group differences between prime categories in ways that would be expected based on the existing literature. Specifically, younger faces were rated as more babyfaced, attractive, and masculine than older faces (*p*s < .001) [1–4]. Also as expected, Black faces were rated as more Afrocentric than White faces (*p* < .001); this effect was not moderated by target age (*p* = .46), indicating that younger and older Black faces did not differ in Afrocentricity. Black faces were also rated as more masculine than White faces (*p* = .001), which aligns with existing findings indicating that Blacks are commonly viewed as more masculine than Whites [5–7].

**Unexpected group differences.** Black faces were rated as more babyfaced and younger than White faces (*p*s < .001), but it seems unlikely that having more neotenous facial features would account for the observed stronger association of Black faces than White faces with danger reported in the main text. Finally, although older faces were rated as more emotionally expressive than younger faces (*p* = .002), all means fell below the scale midpoint of 4.0, suggesting that, overall, the faces were seen as relatively neutral. In addition, prime age did not moderate racial bias in danger associations in either experiment, suggesting that any small difference in emotion expression appears not to have produced a difference in the strength of racial bias as a function of prime age.

# Method

University of Iowa undergraduates (*N* = 266; 222 women, 44 men; *M*_age_ = 19.56 years, *SD*_age_ = 1.88; 79% White) participated for course credit. They completed the online rating study independently on their own time using their personal computers. After consenting to participate, they were randomly assigned to rate all faces from Experiments 1 and 2 (120 faces total) on one of seven attributes (perceived age, perceived race/ethnicity, attractiveness, babyfacedness, masculinity, Afrocentricity, or emotional expressiveness). See Table S1 for attribute item wordings and scales. Faces were presented one-at-a-time in random order within each attribute. After rating all faces, participants completed demographic items and were debriefed.

| **Table S1. Attribute item wordings and response options.** | | |
| --- | --- | --- |
| Attribute | Item Wording | Response Options |
| Age | How old is this person, in years? | Free (numeric) response |
| Race | What is this person's race/ethnicity? | Multiple option response: American Indian or Alaska Native; Asian or Asian American; Black or African American; Hispanic or Latino/a; White; Native Hawaiian or other Pacific Islander; Other |
| Attractiveness | In relation to others of the same race, age, and gender, how attractive is this person? | Rating scale: 1 (*not at all*) to 7 (*extremely*) |
| Babyfacedness | In relation to others of the same race, age, and gender, how babyfaced is this person? | Rating scale: 1 (*not at all*) to 7 (*extremely*) |
| Masculinity | In relation to others of the same race, age, and gender, how masculine is this person? | Rating scale: 1 (*not at all*) to 7 (*extremely*) |
| Afrocentricity | In relation to others of the same race, age, and gender, to what degree does this person have features that are typical of African Americans? | Rating scale: 1 (*not at all*) to 7 (*extremely*) |
| Emotional expressiveness | How emotionally expressive is this person's face? | Rating scale: 1 (*not at all*) to 7 (*extremely*) |

# Analytical approach

We tested whether ratings of face age, race (White and Black), attractiveness, babyfacedness, Afrocentricity, masculinity, or emotional expression differ across the face stimuli used in Experiments 1 and 2.

For each dependent variable, we ran a 2 (Face Age Category: younger vs. older) × 2 (Face Race Category: Black vs. White) × 2 (Experiment: 1 vs. 2) ANOVA. We report means for significant tests.

# Rated Age

The ANOVA produced three main effects (Face Race Category, Face Age Category, and Experiment) plus an interaction of Face Age Category and Experiment.

Anova Table (Type III tests)

 Response: AgeAvg
 Sum Sq Df F value Pr(>F)
 (Intercept) 249994 1 15757.4957 < 2.2e-16 ***
 OldvsYoung 45351 1 2858.5769 < 2.2e-16 ***
 BlackvsWhite 208 1 13.0811 0.0004488 ***
 Experiment 359 1 22.6369 5.880e-06 ***
 OldvsYoung:BlackvsWhite 1 1 0.0380 0.8457453
 OldvsYoung:Experiment 569 1 35.8810 2.593e-08 ***
 BlackvsWhite:Experiment 0 1 0.0048 0.9450059
 OldvsYoung:BlackvsWhite:Experiment 4 1 0.2333 0.6300576
 Residuals 1777 112
 ---
 Signif. codes: 0 '***' 0.001 '**' 0.01 '*' 0.05 '.' 0.1 ' ' 1

### Face Age Category main effect

Older faces were rated as being older than younger faces.

Descriptive statistics for AgeAvg as a function of OldvsYoung.

 OldvsYoung M SD
 Old 67.65 6.14
 Young 27.95 3.48

 Note. M and SD represent mean and standard deviation, respectively.

### Face Race Category main effect

Black faces were rated as being younger than White faces.

Descriptive statistics for AgeAvg as a function of BlackvsWhite.

 BlackvsWhite M SD
 Black 46.41 20.41
 White 49.19 20.76

 Note. M and SD represent mean and standard deviation, respectively.

### Experiment main effect and interaction with Face Age Category

From the pattern of means, older faces in Experiment 1 were rated as being older than older faces in Experiment 2. There was no obvious difference across experiments for younger faces.

Means and standard deviations for AgeAvg as a function of a 2 (OldvsYoung) × 2 (Experiment) design

 Experiment
 1 2
 OldvsYoung M SD M SD
 Old 73.18 3.57 64.89 5.23
 Young 27.32 3.08 28.27 3.66

 Note. M and SD represent mean and standard deviation, respectively.

# Rated Race - Proportion of participants rating face as White

The ANOVA produced main effects of Face Race Category and Experiment plus an interaction of Face Race Category and Experiment. Follow-up means below look at effects of Face Race Category within Experiment.

Anova Table (Type III tests)

 Response: prop_White
 Sum Sq Df F value Pr(>F)
 (Intercept) 24.5524 1 4861.7262 < 2e-16 ***
 OldvsYoung 0.0049 1 0.9605 0.32918
 BlackvsWhite 23.6188 1 4676.8491 < 2e-16 ***
 Experiment 0.0283 1 5.6000 0.01968 *
 OldvsYoung:BlackvsWhite 0.0049 1 0.9605 0.32918
 OldvsYoung:Experiment 0.0094 1 1.8564 0.17578
 BlackvsWhite:Experiment 0.0283 1 5.6000 0.01968 *
 OldvsYoung:BlackvsWhite:Experiment 0.0069 1 1.3719 0.24398
 Residuals 0.5656 112
 ---
 Signif. codes: 0 '***' 0.001 '**' 0.01 '*' 0.05 '.' 0.1 ' ' 1

### Interaction of Face Race Category and Experiment

Categorization of White faces as White was weaker in Experiment 2 (though still > 90%).

Means and standard deviations for prop_White as a function of a 2 (BlackvsWhite) × 2 (Experiment) design

 Experiment
 1 2
 BlackvsWhite M SD M SD
 Black 0.01 0.02 0.01 0.03
 White 0.98 0.02 0.92 0.12

 Note. M and SD represent mean and standard deviation, respectively.

# Rated Race - Proportion of participants rating face as Black

The ANOVA produced a main effect of Face Race Category and an interaction of Face Age Category and Face Race Category.

Anova Table (Type III tests)

 Response: prop_Black
 Sum Sq Df F value Pr(>F)
 (Intercept) 25.1452 1 12760.1983 < 2e-16 ***
 OldvsYoung 0.0066 1 3.3737 0.06890 .
 BlackvsWhite 24.8733 1 12622.2512 < 2e-16 ***
 Experiment 0.0004 1 0.2109 0.64699
 OldvsYoung:BlackvsWhite 0.0118 1 5.9977 0.01587 *
 OldvsYoung:Experiment 0.0037 1 1.8977 0.17108
 BlackvsWhite:Experiment 0.0000 1 0.0234 0.87862
 OldvsYoung:BlackvsWhite:Experiment 0.0012 1 0.5857 0.44569
 Residuals 0.2207 112
 ---
 Signif. codes: 0 '***' 0.001 '**' 0.01 '*' 0.05 '.' 0.1 ' ' 1

### Interaction of Face Age Category and Face Race Category

Categorization of Black faces as Black was weaker for older Black faces than younger Black faces (though still > 90%).

Means and standard deviations for prop_Black as a function of a 2 (BlackvsWhite) × 2 (OldvsYoung) design

 OldvsYoung
 Old Young
 BlackvsWhite M SD M SD
 Black 0.95 0.07 0.98 0.04
 White 0.01 0.03 0.00 0.00

 Note. M and SD represent mean and standard deviation, respectively.

# Rated Attractiveness

The ANOVA produced a main effect of Face Age Category.

Anova Table (Type III tests)

 Response: AttractiveAvg
 Sum Sq Df F value Pr(>F)
 (Intercept) 629.48 1 3235.9320 <2e-16 ***
 OldvsYoung 36.41 1 187.1476 <2e-16 ***
 BlackvsWhite 0.01 1 0.0262 0.8718
 Experiment 0.16 1 0.8402 0.3613
 OldvsYoung:BlackvsWhite 0.18 1 0.9123 0.3416
 OldvsYoung:Experiment 0.02 1 0.0949 0.7586
 BlackvsWhite:Experiment 0.16 1 0.8122 0.3694
 OldvsYoung:BlackvsWhite:Experiment 0.28 1 1.4439 0.2320
 Residuals 21.79 112
 ---
 Signif. codes: 0 '***' 0.001 '**' 0.01 '*' 0.05 '.' 0.1 ' ' 1

### Face Age Category main effect

Younger faces were rated as more attractive than older faces.

Descriptive statistics for AttractiveAvg as a function of OldvsYoung.

 OldvsYoung M SD
 Old 1.84 0.28
 Young 3.00 0.55

 Note. M and SD represent mean and standard deviation, respectively.

# Rated Babyfacedness

The ANOVA produced main effects of Face Age Category and Face Race Category.

Anova Table (Type III tests)

 Response: BabyfaceAvg
 Sum Sq Df F value Pr(>F)
 (Intercept) 824.03 1 3573.6351 < 2.2e-16 ***
 OldvsYoung 47.12 1 204.3459 < 2.2e-16 ***
 BlackvsWhite 4.18 1 18.1100 4.348e-05 ***
 Experiment 0.05 1 0.2082 0.64903
 OldvsYoung:BlackvsWhite 0.48 1 2.0933 0.15074
 OldvsYoung:Experiment 0.70 1 3.0168 0.08515 .
 BlackvsWhite:Experiment 0.68 1 2.9679 0.08769 .
 OldvsYoung:BlackvsWhite:Experiment 0.00 1 0.0001 0.99155
 Residuals 25.83 112
 ---
 Signif. codes: 0 '***' 0.001 '**' 0.01 '*' 0.05 '.' 0.1 ' ' 1

### Face Age Category main effect

Younger faces were rated as more babyfaced than older faces.

Descriptive statistics for BabyfaceAvg as a function of OldvsYoung.

 OldvsYoung M SD
 Old 2.13 0.41
 Young 3.41 0.64

 Note. M and SD represent mean and standard deviation, respectively.

### Face Race Category main effect

Black faces were rated as more babyfaced than White faces.

Descriptive statistics for BabyfaceAvg as a function of BlackvsWhite.

 BlackvsWhite M SD
 Black 3.00 0.88
 White 2.55 0.73

 Note. M and SD represent mean and standard deviation, respectively.

# Rated Masculinity

The ANOVA produced main effects of Face Age Category, Face Race Category, and Experiment.

Anova Table (Type III tests)

 Response: MascAvg
 Sum Sq Df F value Pr(>F)
 (Intercept) 2194.24 1 12078.7492 < 2.2e-16 ***
 OldvsYoung 5.82 1 32.0285 1.184e-07 ***
 BlackvsWhite 1.95 1 10.7326 0.001402 **
 Experiment 1.21 1 6.6697 0.011095 *
 OldvsYoung:BlackvsWhite 0.01 1 0.0280 0.867365
 OldvsYoung:Experiment 0.01 1 0.0430 0.836195
 BlackvsWhite:Experiment 0.06 1 0.3574 0.551166
 OldvsYoung:BlackvsWhite:Experiment 0.02 1 0.0966 0.756480
 Residuals 20.35 112
 ---
 Signif. codes: 0 '***' 0.001 '**' 0.01 '*' 0.05 '.' 0.1 ' ' 1

### Face Age Category main effect

Younger faces were rated as more masculine than older faces.

Descriptive statistics for MascAvg as a function of OldvsYoung.

 OldvsYoung M SD
 Old 4.33 0.43
 Young 4.81 0.47

 Note. M and SD represent mean and standard deviation, respectively.

### Face Race Category main effect

Black faces were rated as more masculine than White faces.

Descriptive statistics for MascAvg as a function of BlackvsWhite.

 BlackvsWhite M SD
 Black 4.71 0.52
 White 4.43 0.46

 Note. M and SD represent mean and standard deviation, respectively.

### Experiment main effect

Experiment 2 faces were rated as more masculine than Experiment 1 faces.

Descriptive statistics for MascAvg as a function of Experiment.

 Experiment M SD
 1 4.43 0.49
 2 4.64 0.50

 Note. M and SD represent mean and standard deviation, respectively.

# Rated Afrocentricity

The ANOVA produced a main effect of Face Race Category.

Anova Table (Type III tests)

 Response: AfrocentricAvg
 Sum Sq Df F value Pr(>F)
 (Intercept) 1438.86 1 15699.0067 <2e-16 ***
 OldvsYoung 0.16 1 1.7028 0.1946
 BlackvsWhite 438.70 1 4786.5837 <2e-16 ***
 Experiment 0.15 1 1.6308 0.2042
 OldvsYoung:BlackvsWhite 0.05 1 0.5537 0.4584
 OldvsYoung:Experiment 0.08 1 0.8420 0.3608
 BlackvsWhite:Experiment 0.23 1 2.4687 0.1190
 OldvsYoung:BlackvsWhite:Experiment 0.11 1 1.2359 0.2686
 Residuals 10.27 112
 ---
 Signif. codes: 0 '***' 0.001 '**' 0.01 '*' 0.05 '.' 0.1 ' ' 1

### Face Race Category main effect

Black faces were rated as more Afrocentric than White faces.

Descriptive statistics for AfrocentricAvg as a function of BlackvsWhite.

 BlackvsWhite M SD
 Black 5.70 0.36
 White 1.67 0.24

 Note. M and SD represent mean and standard deviation, respectively.

# Rated Emotional Expressiveness

The ANOVA produced a main effect of Face Age Category and a 3-way interaction. [Note that the variable is labeled “neutral”, but the items measured emotional expressiveness. Thus, lower scores indicate greater neutrality, higher scores greater emotional expressiveness.]

Anova Table (Type III tests)

 Response: NeutralAvg
 Sum Sq Df F value Pr(>F)
 (Intercept) 893.81 1 3086.0516 < 2.2e-16 ***
 OldvsYoung 2.85 1 9.8435 0.002178 **
 BlackvsWhite 0.45 1 1.5623 0.213932
 Experiment 0.09 1 0.3086 0.579645
 OldvsYoung:BlackvsWhite 0.53 1 1.8421 0.177433
 OldvsYoung:Experiment 0.50 1 1.7407 0.189738
 BlackvsWhite:Experiment 0.01 1 0.0193 0.889794
 OldvsYoung:BlackvsWhite:Experiment 1.28 1 4.4190 0.037782 *
 Residuals 32.44 112
 ---
 Signif. codes: 0 '***' 0.001 '**' 0.01 '*' 0.05 '.' 0.1 ' ' 1

### Face Age Category main effect

Older faces were rated as more emotionally expressive than younger faces. Note that all means are below the scale midpoint of 4.

Descriptive statistics for NeutralAvg as a function of OldvsYoung.

 OldvsYoung M SD
 Old 3.07 0.61
 Young 2.70 0.47

 Note. M and SD represent mean and standard deviation, respectively.

### Within Experiment 1: Face Age Category × Face Race Category

From the pattern of means, younger White faces were rated as not particularly emotionally expressive; older White faces were rated the most emotionally expressive.

Means and standard deviations for NeutralAvg as a function of a 2 (OldvsYoung) × 2 (BlackvsWhite) design

 BlackvsWhite
 Black White
 OldvsYoung M SD M SD
 Old 2.91 0.49 3.13 0.49
 Young 3.08 0.61 2.58 0.51

 Note. M and SD represent mean and standard deviation, respectively.

### Within Experiment 2: Face Age Category × Face Race Category

From the pattern of means, younger White AND Black faces were rated as less emotionally expressive than older faces; older Black faces were rated the most emotionally expressive.

Means and standard deviations for NeutralAvg as a function of a 2 (OldvsYoung) × 2 (BlackvsWhite) design

 BlackvsWhite
 Black White
 OldvsYoung M SD M SD
 Old 3.19 0.73 3.00 0.60
 Young 2.65 0.29 2.61 0.45

 Note. M and SD represent mean and standard deviation, respectively.

# References

1. Deuisch FM, Zalenski CM, Clark ME. Is there a double standard of aging? J Appl Soc Psychol. 1986;16:771-85.
2. Kite ME, Deaux K, Miele M. Stereotypes of young and old: Does age outweigh gender? Psychol Aging. 1991;6:19-27.
3. Kite ME, Stockdale GD, Whitley BE, Johnson BT. Attitudes toward younger and older adults: An updated meta‐analytic review. J Soc Issues. 2005;61:241-66.
4. O’Connell AN, Rotter NG. The influence of stimulus age and sex on person perception. J Gerontol. 1979;34:220-228.
5. Galinsky AD, Hall EV, Cuddy AJ. Gendered races: Implications for interracial marriage, leadership selection, and athletic participation. Psychol Sci. 2013;24:498-506.
6. Goff PA, Thomas MA, Jackson MC. “Ain’t I a woman?”: Towards an intersectional approach to person perception and group-based harms. Sex Roles. 2008;392-403.
7. Johnson KL, Freeman JB, Pauker K. Race is gendered: How covarying phenotypes and stereotypes bias sex categorization. J Pers Soc Psychol. 2012;102:116-31.
