# Supplementary material for: Racial bias in implicit danger associations generalizes to older male targets
Source: PLoS One. 2018 Jun 6;13(6):e0197398. doi: 10.1371/journal.pone.0197398 (PMC5991338; doi:10.1371/journal.pone.0197398)
Supplement: S2 Supporting Information — (DOCX) [file pone.0197398.s002.docx]

S2 Supporting Information

Facial Stimuli Selection Procedure in Experiment 2

# Method

**Face stimuli categories.** As reported in the main text, face stimuli for Experiment 2 were obtained from the Florida Department of Corrections website (<http://www.dc.state.fl.us/>). This database is publicly available online, maintains a relative degree of experimental control (e.g., head-and-shoulder photographs taken against a blue background), and, importantly, information relevant to Experiments 1 and 2 (age and race) was provided for each inmate. We aimed to gather 20 stimuli in each of four age/race combinations: older Black, older White, younger Black, and younger White. For this experiment, “young” referred to men listed on the database as between the ages of 18 and 35, and “old” referred to men listed as over the age of 70. Stimuli were also selected based on their race, as listed on the database (Black or White).

**Random selection procedure**. To avoid potential selection biases on our part, we used a randomized selection procedure to select the images. First, we used a random number generator to select a specific page in the database. Next, we generated another random number to select a specific image on that page. If this image depicted a man who did not meet our age or race criteria or a man with glasses, tattoos, or other idiosyncrasies (e.g., scars), the image was not used, and the random selection procedure was carried out again until we found an image that fit our criteria. We repeared this procedure until we had 20 images that met our criteria for each of the four age/race groups, resulting in 80 new face stimuli.
